# Supplementary figures and images for: Uncovering the mechanism of anthocyanin accumulation in a purple-leaved variety of foxtail millet (Setaria italica) by transcriptome analysis
Source: PeerJ. 2022 Oct 3;10:e14099. doi: 10.7717/peerj.14099 (PMC9536322; doi:10.7717/peerj.14099)

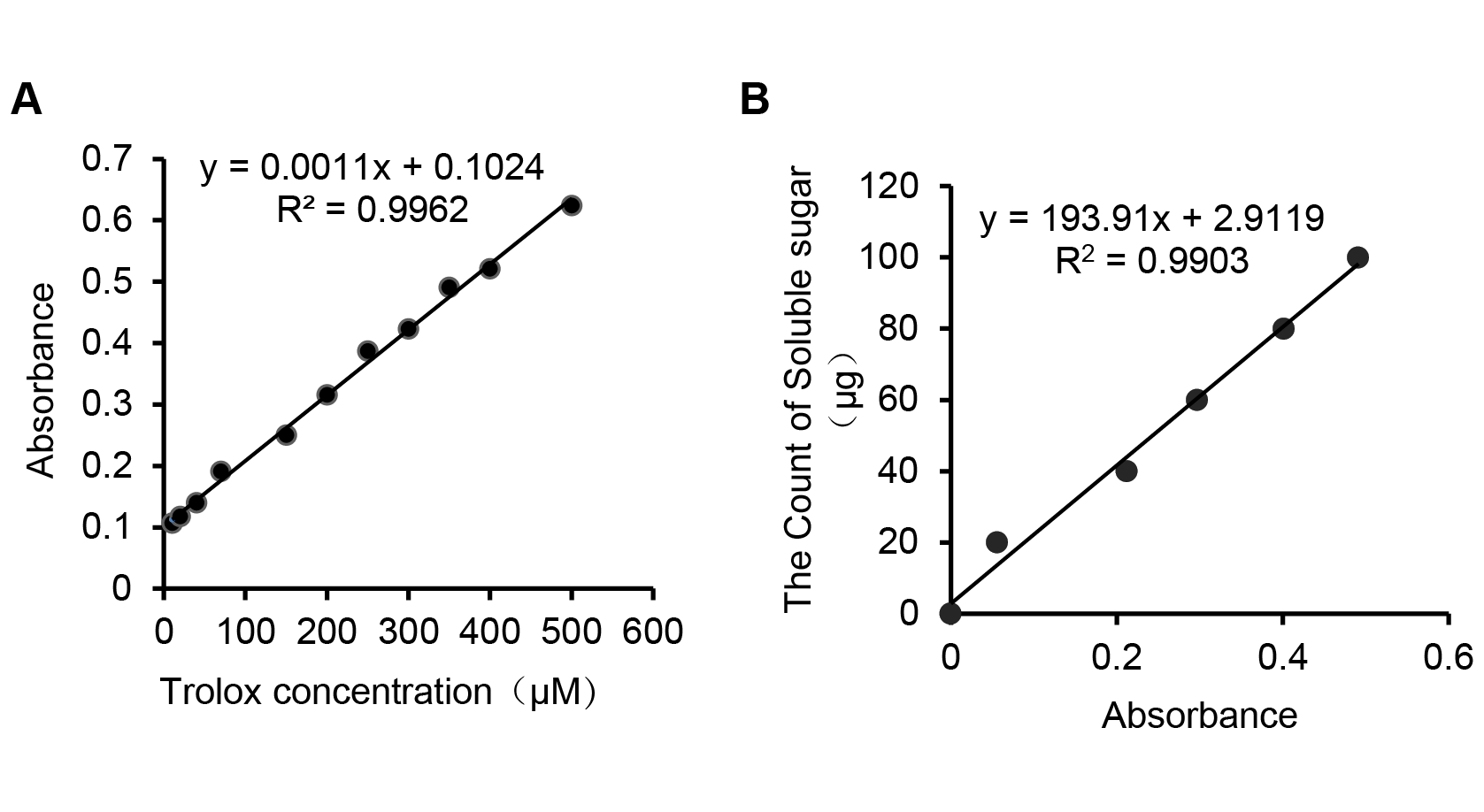

Supplement: Supplemental Information 1 — (A) The standard curve of total antioxidant capacity analysis by Ferric-reducing Antioxidant Power (FRAP) method. (B) The standard curve of total soluble sugar measurement. [file peerj-10-14099-s001.png]

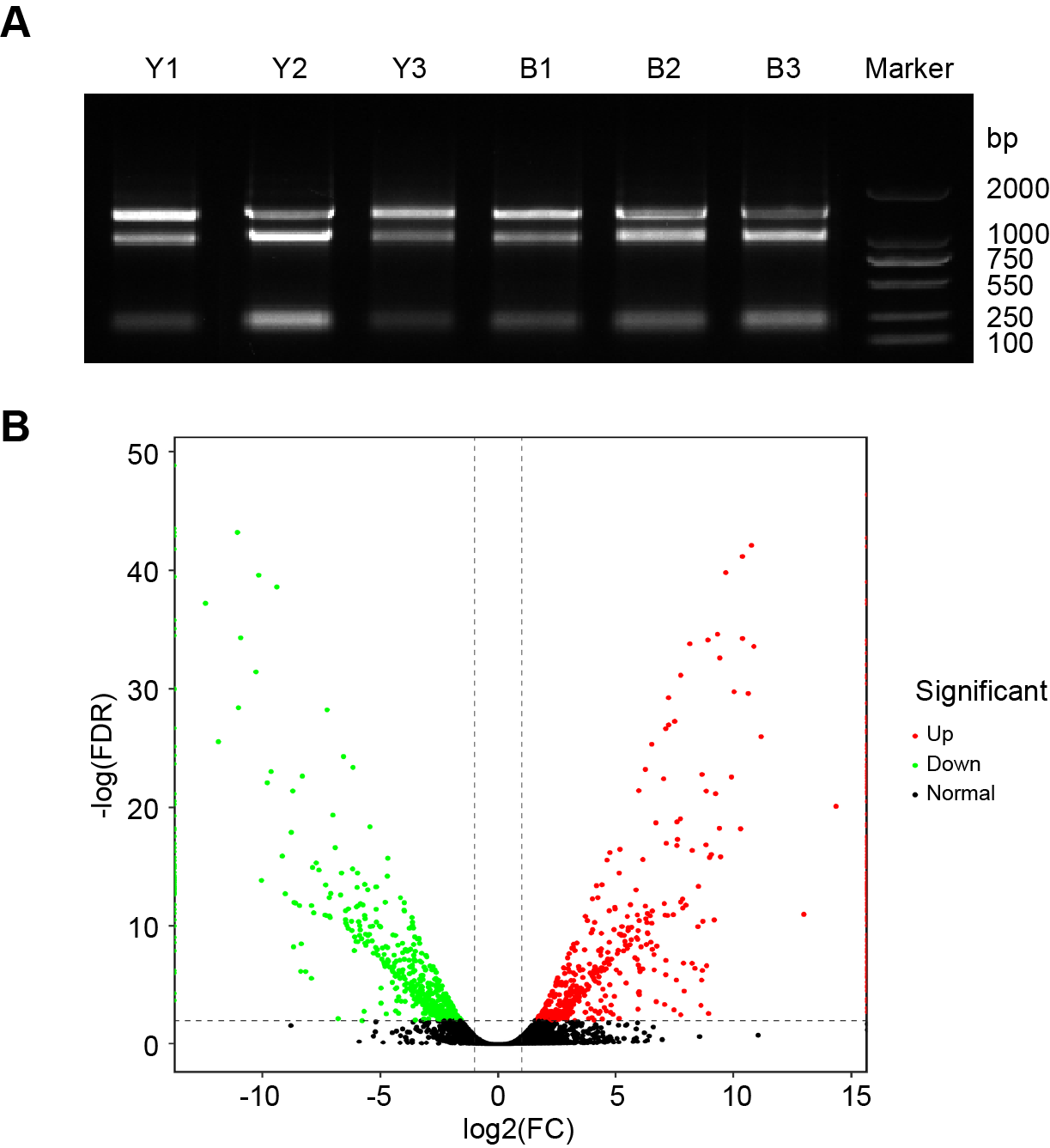

Supplement: Supplemental Information 2 — (A) RNA for sequencing was detected through agarose gel electrophoresis. (B) Analysis of DEGs. Each point represents a gene, and the horizontal coordinate represents the log value of the multiple difference between the expression levels of a gene in the two samples. The vertical axis represents the negative log of the statistical significance of the change in gene expression. [file peerj-10-14099-s002.png]

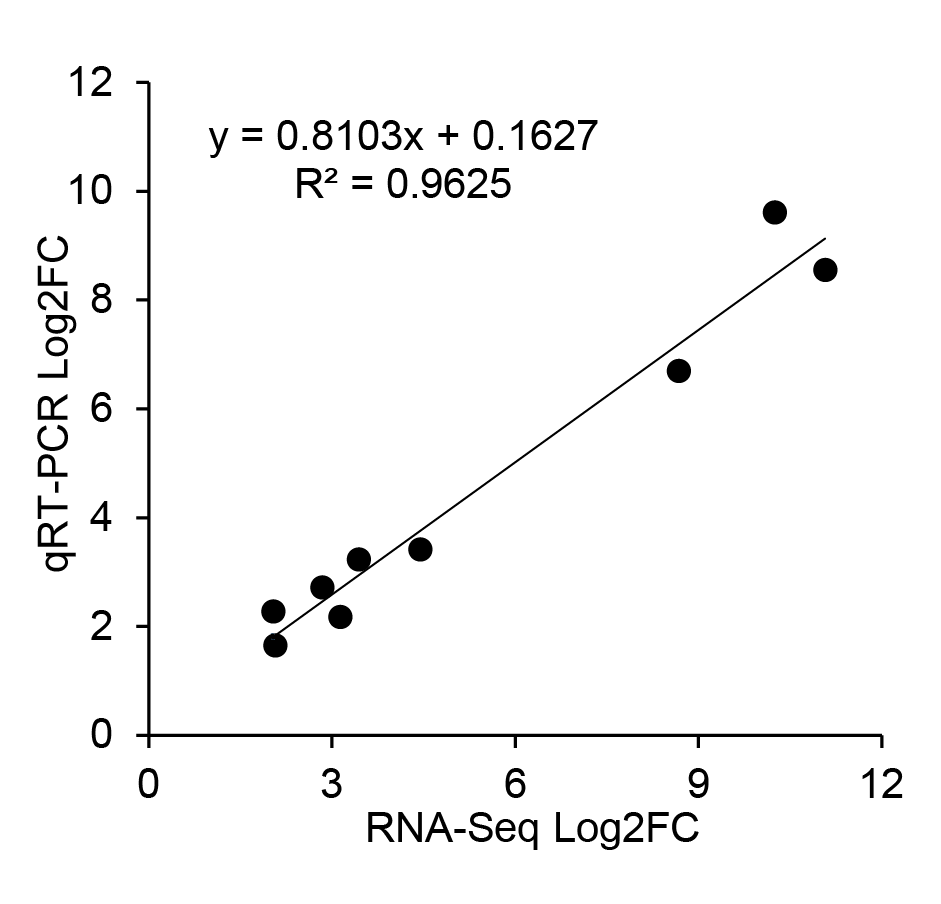

Supplement: Supplemental Information 3 — The values on the abscissa and ordinate represent the multiple differences in the expression levels of 9 genes verified by RNA-sequencing and qRT-PCR, respectively. [file peerj-10-14099-s003.png]
