# Supplementary material for: Uncovering the mechanism of anthocyanin accumulation in a purple-leaved variety of foxtail millet (Setaria italica) by transcriptome analysis
Source: PeerJ. 2022 Oct 3;10:e14099. doi: 10.7717/peerj.14099 (PMC9536322; doi:10.7717/peerj.14099)
Supplement: Table S1 [file peerj-10-14099-s004.pdf]

**Supplemental Table 1: Summary and Quality Control Items of Samples for RNA-sequencing**

| Sample | Clean reads | Clean bases | GC content (%) | ≥Q30 percentage (%) |
|---|---|---|---|---|
| Y1 | 21,324,300 | 6,397,290,000 | 58.72% | 94.43% |
| Y2 | 28,070,803 | 8,421,240,900 | 58.59% | 93.08% |
| Y3 | 24,245,204 | 7,273,561,200 | 59.01% | 93.54% |
| B1 | 21,970,319 | 6,591,095,700 | 58.24% | 92.84% |
| B2 | 26,008,340 | 7,802,502,000 | 58.41% | 92.98% |
| B3 | 23,058,580 | 6,917,574,000 | 58.76% | 93.14% |
