# Supplementary material for: Uncovering the mechanism of anthocyanin accumulation in a purple-leaved variety of foxtail millet (Setaria italica) by transcriptome analysis
Source: PeerJ. 2022 Oct 3;10:e14099. doi: 10.7717/peerj.14099 (PMC9536322; doi:10.7717/peerj.14099)
Supplement: Table S2 [file peerj-10-14099-s005.pdf]

Supplemental Table 2: Summary for Comparative Efficiency

Sample	Total Reads	Mapped Reads（%）	Uniq Mapped Reads	Multiple Map Reads	Reads Map to ‘+’	Reads Map to ‘-’
Y1	42,648,600	38,823,519 (91.03%)	38,164,899 (89.49%)	658,620 (1.54%)	19,302,972 (45.26%)	19,389,585 (45.46%)
Y2	56,141,606	51,370,743 (91.50%)	50,404,721 (89.78%)	966,022 (1.72%)	25,552,998 (45.52%)	25,666,496 (45.72%)
Y3	48,490,408	43,823,831 (90.38%)	43,047,230 (88.77%)	776,601 (1.60%)	21,810,544 (44.98%)	21,888,593 (45.14%)
B1	43,940,638	39,072,277 (88.92%)	38,449,360 (87.50%)	622,917 (1.42%)	19,441,124 (44.24%)	19,530,634 (44.45%)
B2	52,016,680	46,801,329 (89.97%)	46,034,830 (88.50%)	766,499 (1.47%)	23,291,964 (44.78%)	23,391,083 (44.97%)
B3	46,117,160	40,605,203 (88.05%)	39,948,835 (86.62%)	656,368 (1.42%)	20,210,469 (43.82%)	20,297,111 (44.01%)
