# Supplementary material for: Uncovering the mechanism of anthocyanin accumulation in a purple-leaved variety of foxtail millet (Setaria italica) by transcriptome analysis
Source: PeerJ. 2022 Oct 3;10:e14099. doi: 10.7717/peerj.14099 (PMC9536322; doi:10.7717/peerj.14099)
Supplement: Table S3 [file peerj-10-14099-s006.pdf]

**Supplemental Table 3: Primer for qRT-PCR Used in This Study**

| Genes | Accession | Primer (5’-3’) | Amplicon length (bp) |
|---|---|---|---|
| *PAL* | Seita.1G240200 | F:TCCCGCTGTACCGCTTCGT<br>R:CCCACTCCTTGAGGCACTCG | 153 |
| *4CL* | Seita.9G537200 | F:ATTTACAATCAAGGAAGGGTGG<br>R:AGGAAACGGAATAACAACAGC | 190 |
| *DFR* | Seita.5G237900 | F:ATCCTCAAGCAGGTGCAGTTCG<br>R:CCGTGGATGGTGGAGTCGTG | 122 |
| *LDOX-1* | Seita.4G054600 | F:GGAGCCTATCCCAATCAT<br>R:CGCTTGCCGTCTATCAG | 213 |
| *LDOX-2* | Seita.1G000700 | F:CACGAACACGACGACGACCTCC<br>R:GGCACGCCGTTGTGGATGA | 137 |
| *UFGT* | Seita.3G190000 | F:TTTCCAAGCCCAGATATACGG<br>R:GAAAGTGTTGACGGCGATTC | 138 |
| *5GT* | Seita.2G324600 | F:TCTGTACGCCGAGCAGCACC<br>R:TTCCGTCTCGCCCATCAGG | 154 |
| *GT* | Seita.5G239500 | F:TTCGGCACGCTGTCCCATTT<br>R:ACCCGTCGGGCAACCACTCT | 133 |
| *AT* | Seita.9G002300 | F:CCCTCCCGCTAACCTTCTTTG<br>R:TCGTAGGAGACGAGGTCATCCA | 280 |
| *Actin* | Seita.5G464000 | F:TGCTCAGTGGAGGCTCAACA<br>R:CCAGACACTGTACTTGCGCTC | 128 |
